# Prognostic Values of BolA Family Member Expression in Hepatocellular Carcinoma

**DOI:** 10.1155/2022/8360481

**Published:** 2022-08-16

**Authors:** Dong Wang, ZhiMing Wang, YiMing Tao

**Affiliations:** ^1^Department of Liver Disease Center, The Affiliated Hospital of Qingdao University, Qingdao, Shandong, China; ^2^Department of General Surgery, Xiangya Hospital, Central South University, Changsha, Hunan, China

## Abstract

The BolA gene family member (BOLA1–3) plays an important role in regulating normal and pathological biological processes including liver tumorigenesis. However, their expression patterns as prognostic factors in hepatocellular carcinoma (HCC) patients have not to be elucidated. We examined the transcriptional expressions and survival data of BolA family member in patients with HCC from online databases including ONCOMINE, TCGA, UALCAN, Gene Expression Profiling Interactive Analysis (GEPIA), Kaplan-Meier plotter, SurvExpress, cBioPortal, and Exobase. Network molecular interaction views of BolA family members and their neighborhoods were constructed by the IntAct web server. In our research, we had found that the expression levels of BolA /2/3 mRNA were higher in HCC tissue than in normal liver tissues from TGCA databases. Moreover, the BolA family gene expression level is significantly associated with distinct tumor pathological grade, TMN stage, and overall survival (OS). The BolA family can be considered as prognostic risk biomarkers of HCC. A small number of BolA gene-mutated samples were detected in the HCC tissue. IntAct analysis revealed that BolA1/2/3 was closely associated with the GLRX3 expression in HCC, which is implicated in the regulation of the cellular iron homeostasis and tumor growth. Furthermore, prognostic values of altered BolAs and their neighbor GLRX3 gene in HCC patients were validated by SurvExpress analysis. In conclusion, the membrane BolA family identified in this study provides very useful information for the mechanism of hepatic tumorigenesis.

## 1. Introduction

Hepatocellular carcinoma (HCC) has very aggressive neoplasms and describes as a major health problem worldwide [1]. Genetic and epigenetic alterations, which lead to uncontrolled cellular proliferation and metastasis, are the characters of HCC development.

Recent research has revealed a critical role for cellular iron homeostasis in the clinical context of liver tumorigenesis [2, 3]. Although significant progress has been made in understanding the iron homeostasis disruption associated with HCC, the precise molecular signals that trigger initiation and progression of HCC remain to be identified.

The human BolA gene family consists of BOLA1, BOLA2, and BOLA3 [4]. It has been suggested that BolA family members serve as assembly factors for mitochondrial iron-sulfur (Fe/S) cluster proteins that has involvement in cancer cell biology [5, 6]; although, the functions of BOLA1 and BOLA3 are still undefined in cancer. Prior research has highlighted the importance role of BOLA3 in human endothelial metabolism and cardiovascular disease pathogenesis [7]. More specifically, evidence points out that BOLA2 has been shown to be highly correlated with hepatic iron homeostasis [8]. And yet, even the overexpression of BOLA2 is required to drive HCC tumor growth and tumor hemorrhage [9, 10], and high BOLA2 can promote tumor growth and predict the HCC prognosis [11]. BOLA1 plays a leading role in mitochondrial morphology by potential regulation and can induce diseases [12]. In the ovarian cancer, the BOLA2 and BOLA3 were higher in cancer tissues and may act as prognostic biomarkers [13], and in the lung adenocarcinoma, the BOLA3 was correlated with the immune cell infiltrates [14]. However, it has been poorly characterized whether the expression of BolA family members in HCC is correlated with clinical outcomes.

In our research, we analyzed the BolA family member mRNA level in HCC tissues and nontumor liver tissues by the public database. In addition, we investigated correlation between their expressions and clinical characteristics and performed SurvExpress analysis of prognostic risks for overall survival. The results showed that BOLA1\2\3 may be a promising biomarker for the prognosis in HCC.

## 2. Material and Methods

### 2.1. ONCOMINE Database Analysis

The difference mRNA expression level of the BolA family gene in human cancer was identified in the ONCOMINE online microarray database (http://www.oncomine.org). For each BolA family gene, the thresholds were set as the following values: *P* value of 0.01, fold change of 2, and gene ranking of all. Analysis type was set as follows: cancer vs. normal analysis.

### 2.2. UALCAN Database Analysis

The UALCAN online database (http://ualcan.path.uab.edu) was used to calculate the BolA gene expression level and clinicopathologic parameters in the TCGA database on patient with LIHC (liver hepatocellular carcinoma) [15] .

### 2.3. cBioPortal and Exobase Database Analysis

The cBio Cancer Genomics Portal (http://www.cbioportal.org/) performed to estimate the cancer genomics data sets of BolA family gene using TCGA-LIHC data [16]. The exoRBase database (http://www.exoRBase.org) can analysis the human blood exosomes, including circRNA, lncRNA, and mRNA [17].

### 2.4. GEPIA Database Analysis

Gene Expression Profiling Interactive Analysis (GEPIA) web server (http://gepia.cancer-pku.cn/) was used to study the correlation mRNA expression of BolA family members and overall survival (OS) in LIHC [18]. A total of 331 LIHC patients were enrolled, and “median” was regarded as group cutoff value.

### 2.5. Kaplan-Meier Plotter Analysis

The Kaplan-Meier (KM) plotter database (http://kmplot.com/analysis) was used to calculate the survival time in LIHC patients [19]. Briefly, each BolA family member was individually analyzed to obtain KM plots. Group cutoff was set as “median.” Hazard ratios (HR) with 95% confidence intervals (CI) were extracted from the KM plotter webpage. Overall survival (OS) data from 364 patients with HCC were enrolled.

### 2.6. SurvExpress Database Analysis

SurvExpress (http://bioinformatica.mty.itesm.mx/SurvExpress) was used for obtaining survival data for the expression of BolA family members in patient with LIHC, for which information was not available on the GEPIA and KM plotter database [20]. Briefly, in the TCGA-LIHC datasets containing 381 samples, BOLA1, BOLA2, and BOLA3 were entered into the number-at-risk cases, median mRNA expression levels, HRs, 95% confidence interval (CI), and *P* values that were displayed.

### 2.7. IntAct Database Analysis

IntAct (http://www.ebi.ac.uk/intact) was applied to identify densely connected network components and BolA family members, for which protein-protein interaction enrichment analysis data populated by either curated from the literature or from direct data depositions [21].

### 2.8. Western Blot Analysis

Western blot analysis was performed as previously described [11]. The antibody dilutions were 1 : 1,000 for BOLA1 polyclonal antibody (Cat. # 18017-1-AP, Proteintech), 1 : 1,000 for BOLA2 polyclonal antibody (Cat. # ab169481, Abcam), 1 : 1,000 for BOLA3 polyclonal antibody (Cat. # ab185339, Abcam), and 1 : 5,000 for the *β*-actin mouse monoclonal antibody (Sigma-Aldrich, Cat. # A1978).

## 3. Results

### 3.1. BolA Family Members Are Frequently Upregulated in HCC

**Figure 1:**
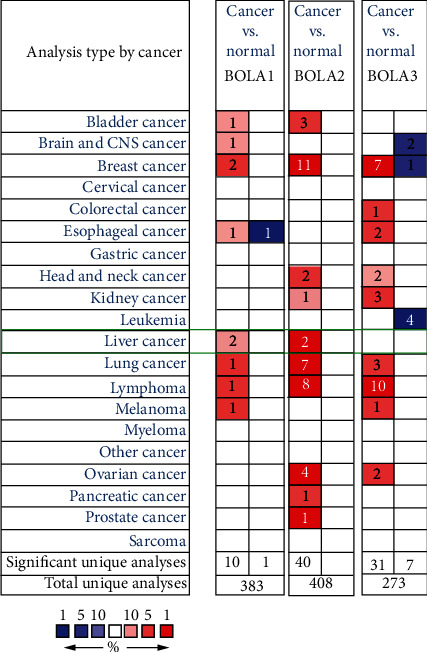
Transcriptional expression of BolA family members in 20 different types of cancer types (ONCOMINE database). Notes: the BOLA1\2\3 mRNA expression (cancer tissue vs. normal tissue) was compared by Students' *t*-test. Cut-off of change values was as follows: *P* value: 0.01, fold change: 1.5, gene rank: 10%, and data type: mRNA.

In order to analysis the expression differences of the BolA family, we first performed an analysis using the ONCOMINE database to investigate differences in the mRNA levels of each BolA family in cancers. As shown in Figure 1, the number of the upregulation BOLA1\2\3 expression was found in tumors compared with normal tissues in various types of cancers. Significantly higher mRNA expressions of BOLA1\2 were found in multiple HCC tissues datasets. The BOLA1\2 overexpression was found in HCC tissues compared with normal tissues in Roessler Liver 2 dataset (1.51-fold increase, *P* = 3.26*E* − 33; 2.67-fold increase, *P* = 3.72*E* − 83, respectively) [22], while were observed in Wurmbach liver dataset (1.65-fold increase, *P* = 0.003; 2.21-fold increase, *P* = 4.67*E* − 4, respectively) [23]. Significant upregulation of BOLA1\2 was also found in Chen Liver dataset (1.57-fold increase, *P* = 1.00*E* − 8; 1.56-fold increase, *P* = 2.78*E* − 13, respectively) [24]. The abovementioned observations suggest that the overexpression of BOLA family members is associated with cancer progression and might be of clinical importance.

### 3.2. BolA Family Member Expression Was Higher in HCC

**Figure 2:**
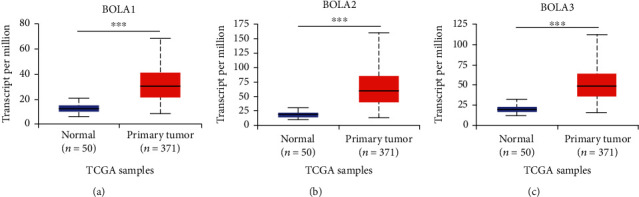
The BOLA1\2\3 mRNA expressions in HCC and adjacent nontumorous tissues (UALCAN database). Notes: BolA family gene mRNA was higher in HCC tissues compared to nontumorous tissues. Statistically significant changes were indicated with asterisks. ^∗∗∗^*P* < 0.001.

**Figure 3:**
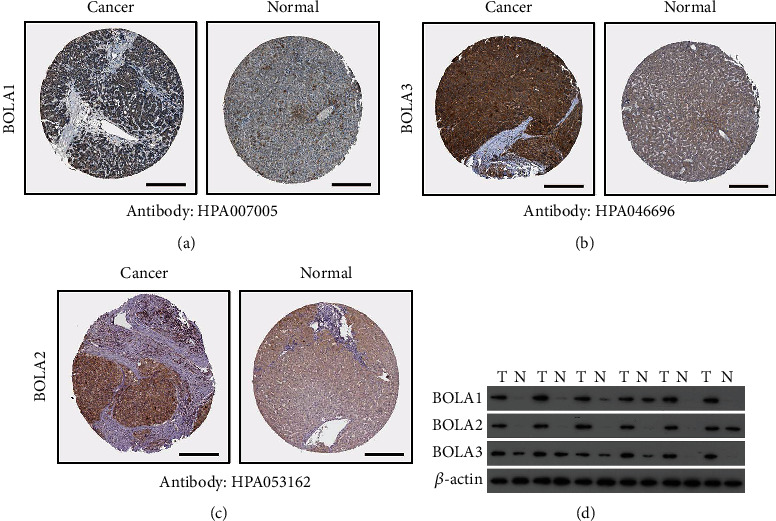
Protein expressions of BolA family members in human normal liver tissue and HCC (Human Protein Atlas database). Notes: BOLA1/2/3 proteins were lower in normal liver tissues than in HCC tissues. BOLA1 antibody HPA007005, BOLA2 antibody HPA046696, and BOLA3 antibody HPA053162. Scale bar is 100 *μ*m (a)–(c). (d) The BOLA1/2/3 expression in the HCC and nontumor samples was as follows, and we had found that the BOLA1/2/3 BOLA1\2\3 expression level was higher in HCC than in the nontumor. T: tumor; NT: nontumor.

To further validate the observations made in the ONCOMINE database, TCGA-LIHC cohort performed a retrospective study. As shown in Figure 2, the BolA family expression level in HCC was higher than in the normal liver tissues (*P* < 0.05). In order to confirm this, we investigated protein levels of BolA family members by the Human Protein Atlas database (http://www.proteinatlas.org/pathology) [25]. As shown in Figure 3, BOLA1\2\3 proteins had lower level in the normal liver, while medium and high level were observed in HCC. And we also found that the BOLA1\2\3 expression level was higher in HCC than in the nontumor using our HCC samples. Human BolA proteins (BOLA1\2\3) are novel nonclassical secreted proteins [4]. In addition, a very low mutation rate of BOLA1\2\3 was observed in HCC patients (Figure S1A), the BOLA1 mutation rate was 4%, and there was no mutation in BOLA3. Intriguingly, using the Exosomes web-accessible database (http://www.exoRBase.org) analysis, the increased expression of BOLA2 may be used as circulating biomarkers for HCC patients (Figure S1B). Taken together, BOLA2 may had the potential ability for HCC diagnose.

### 3.3. Association between BolA Family Member and Tumor Grades and Stages

**Figure 4:**
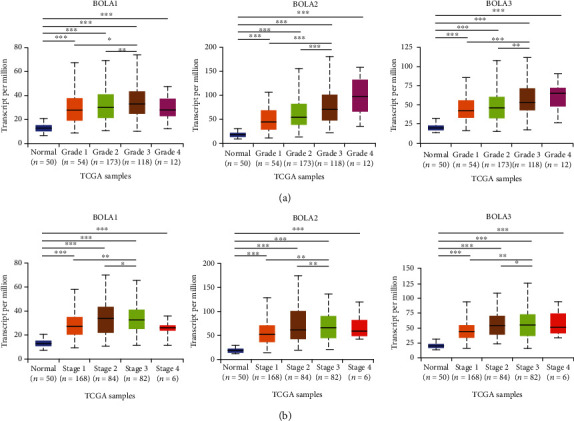
BolA family member mRNA expressions with clinicopathologic parameters in HCC (UALCAN database). Notes: mRNA expressions of BolA family members were significantly related to tumor grades, and as tumor grade increased, the mRAN expressions of BolAs tended to be higher (a). mRNA expressions of BolA family members were remarkably correlated with clinical stages, and patients who were in more advanced stages tended to express higher mRNA expression of BolAs (b). Data are mean ± SEM. ^∗^*P* < 0.05; ^∗∗^*P* < 0.01; ^∗∗∗^*P* < 0.001.

Both the mRNA and protein expression of BolA family members were found to be overexpressed in HCC; we next analyzed the relationship between mRNA expressions of each BolA family members with clinicopathological parameters of HCC patients by UALCAN. As was shown in Figure 4(a), we found that the elevated level of BOLA1\2\3 mRNA had a higher proportion of high-grade tumors (G3/G4). The BOLA1\2\3 mRNA level had significantly correlated with tumor stage in HCCs, which means that the advanced stage HCCs can express higher BolA mRNA (Figure 4(b)). The reason why mRNA expressions of BOLA1\2\3 in stage 3 seemed to be higher than that in stage 4 may be due to the small sample size (only 6 HCC patients were at stage 4). These findings indicated that the BOLA1\2\3 may accelerate HCC growth and progression.

### 3.4. BolA Family Member Predicts the Prognosis in HCC Patients

**Figure 5:**
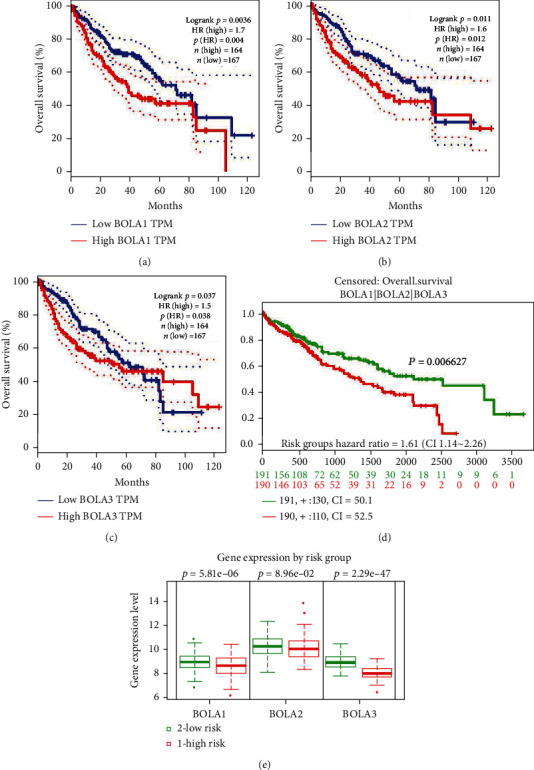
The BolA family members with clinical outcomes in HCC patients by Kaplan-Meier curves (GEPIA database and SurvExpress database). Notes: overall survival data of BolA family members are generated from the GEPIA web server (a)–(c). Prognostic risk of the mRNA expression of BolA family members in HCC patients (d). The concordance index and *P* value of log-rank testing equality of survival curves are indicated. The box plots indicate the difference in the expression of gene between risks groups, and *P* values are derived from *t*-test between both groups (e).

We used GEPIA web server to analyze the prognostic values of BolAs in TCGA-LIHC patients. As were shown in Figure 5, upregulation of BOLA1, BOLA2, and BOLA3 were significantly associated with shorter OS (HR = 1.7, *P* = 0.0036; HR = 1.6, *P* = 0.012; HR = 1.5, *P* = 0.038, respectively, Figures 5(a)–5(c)). The relationship between combinatory mRNA expressions of all 3 BolA family members and prognosis of liver cancer patients were further analyzed by SurvExpress. In our study, we also found that higher combinatory mRNA expressions of all 3 BolA family members were associated with poorer OS in LIHC patients (HR = 1.61, 95% CI: 1.14-2.26, and *P* = 0.006627, Figure 5(d)). And then, we anlayed the prognostic role of BolA family members in HCC patients.. As was shown in Figure 5(e), the higher mRNA expression of BOLA1 (*P* = 5.81*E* − 06), BOLA2 (*P* = 8.96*E* − 02), and BOLA3 (*P* = 2.29*E* − 47) was significantly associated with shorter OS of LIHC patients. These results indicated that mRNA expressions of BOLA1\2\3 may be exploited as useful biomarkers for prediction of HCC patient's survival.

### 3.5. Identification of Hub BolA Family Member and Their Clinical Value in HCC

**Figure 6:**
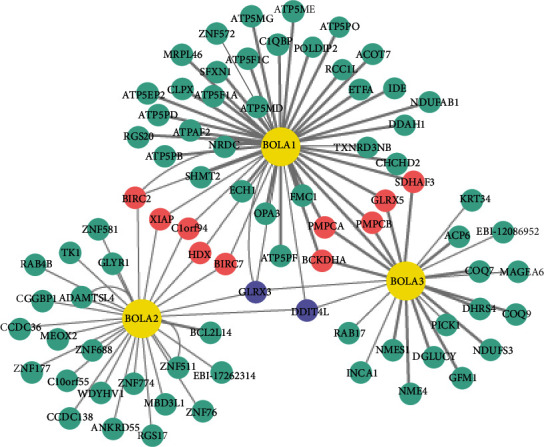
Network protein-protein interaction views of BolA family members and their neighborhood in TCGA-LIHC patients (IntAct database). Notes: network of molecular interaction was constructed by IntAct. Red marker indicates activation relationship of two intermediate related genes, including BIRC2, XIAP, C1orf94, HDX, BIRC7, BCKDHA, PMPCA, PMPCB, GLRX5, and SDHAF3. Purple marker indicates activation relationship of three intermediate related genes, including GLRX3 and DDIT4L.

**Figure 7:**
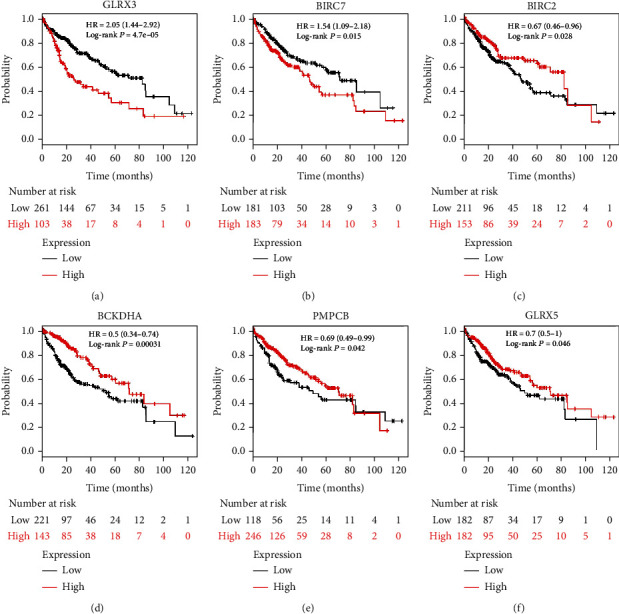
Prognostic values of BolA family altered neighbor genes in HCC patients (Kaplan-Meier plotter). Notes: representative altered neighbor genes of BolA family members, including GLRX3 (a), BIRC7 (b), BIRC2(c), BCKDHA (d), PMPC8 (e), and GLRX5 (f).

**Figure 8:**
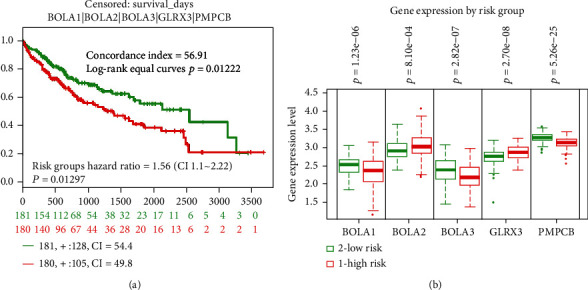
Combinatory BolA family members and GLRX3 and PMPCB predict survival of TCGA-LIHC patients (SurvExpress dataset). Notes: (a) Kaplan–Meier survival curve of 361 TCGA HCC samples using the SurvExpress database, based on the low or high risk for a poor outcome. (b) The high expression of five hub genes is correlated with high risk, poor prognosis, and shorter overall survival time. High- and low-risk groups are labeled with red and green curves, respectively. The box plots indicate the difference in expression of gene between risks groups, and *P* values are derived from *t*-test between both groups.

After analyzing the genetic alterations in BolAs and their prognostic value in HCC patients, we further analyzed the protein-protein interaction network among BolAs using IntAct databases. The top hub genes were GLRX3, DDIT4L, BIRC7, HDX, C1orf94, XIAP, BIRC2, BCKDHA, PMPCA, PMPCB, GLRX5, and SDHAF3 (Figure 6). As was shown in Figure 7, Kaplan-Meier (KM) plotter survival analysis, based on clinical information from the TCGA liver cancer datasets, revealed that the low expression of BIRC2 (HR = 0.67, 95% CI: 0.46-0.96, and *P* = 0.028, Figure 7(c)), BCKDHA (HR = 0.5, 95% CI: 0.34-0.74, and *P* = 0.00031, Figure 7(d)), PMPCB (HR = 0.69, 95% CI: 0.49-0.99, and *P* = 0.042, Figure 7(e)), and GLRX5 (HR = 0.7, 95% CI: 0.5-1, and *P* = 0.046, Figure 7(f)) significantly correlated with shorter OS of LIHC patients. GLRX3 (HR = 2.05, 95% CI: 1.44-2.92, and *P* = 4.7*E* − 5, Figure 7(a)) and BIRC7 (HR = 1.54, 95% CI: 1.09-2.18, and *P* = 0.015, Figure 7(b)) were quite the contrary. Notably, higher combinatory mRNA expressions of BOLA2 with GLRX3 were associated with poorer OS in HCC patients (HR = 1.56, 95% CI: 1.1-2.22, *P* = 8.1*E* − 4 and *P* = 2.7*E* − 8, respectively, Figure 8). Many studies have investigated the expression of GLRX3 imply in regulating HCC cell proliferation, growth, and microvascular invasion via disruption of iron homeostasis [26]. Thus, we could guess that BOLA2 has the ability to promote the development of HCC and maintains cancer cell growth in the condition of metabolic stress.

## 4. Discussion

HCC is one of the leading causes of lethal, and there is great interest in understanding the underlying differentially expressed genes involved in the development and progression of individual tumors. In this study, we investigated the relationship between BolA family members and HCC patients using comprehensive data mining. We found that BolA family members are uniquely overexpressed in HCCs. Moreover, the mRNA expression levels of BolA family genes are associated with distinct tumor grade, TMN stage, and OS. Thus, BOLA1, BOLA2, and BOLA3 can predict the prognosis of HCC patients and may serve as oncogenes that promote HCC growth.

It has been proved that HCC development is a multistep process, including cell proliferation, adhesion, and metabolism. Iron metabolism plays an important role in both normal and cancer cells. In the process of HCC development, more iron is required to maintain the cancer cell proliferation, growth, and self-renewal in stem cells [27]. BOLA1, a mitochondrial protein, makes balances the effect of L-buthionine-(S, R)-sulfoximine (BSO)-induced glutathione (GSH) depletion on the mitochondrial thiol redox potential [12]. BOLA3 plays an important role in form [2Fe-2S] cluster-bridged dimeric heterocomplexes with the human monothiol glutaredoxin GRX5 [28]. A recent study indicated that BOLA1 and BOLA3 are associated with clinical outcomes in many diseases [5]. However, a thoughtful description of the relationship between expression level and cancer prognosis has not been analyzed. Although the increased expression of BOLA1/3 was obverse in present study, a correlation was observed between BOLA1/3 expression and defined genes in LIHC, such as oncogenic activity of BIRC2 [29] and tumor suppressor PMPCB [30]. Therefore, we can speculate that the BOLA1/3 expression in HCCs contributes to uncontrolled cell cellular proliferation. Further studies will be needed to clarify its role in HCC.

BOLA2, a gene associated with iron homeostasis, has been described in its biological function by the animal model [10]. The mechanisms of BOLA2 regulation are as follows: (i) specific in-frame fusion transcript regulation [31], (ii) monothiol CGFS glutaredoxin binding partners, (iii) GRX3-dependent anamorsin maturation pathway [32], and (iv) as c-Myc-regulated gene in HCC [10]. In our study, BOLA2 and GLRX3 are frequently overexpressed in HCC tumors tissues. Interestingly, our study revealed that the upregulation of BOLA2 and GLRX3 was associated with worse OS in patients with HCC. Up to now, more and more novel biomarkers, such as circular RNAs (circRNAs) [33], circulating microRNAs [34], and serum extracellular vesicles [35], had appeared for diagnosing HCC and predicting clinical outcomes. Our study analyzed the relationship between BOLA2 and serum extracellular vesicles. Hence, we postulate that the BOLA2 may have the potential for predicting the prognosis in HCC patients. Due to the limitations in our study, the relationship between the BOLA2 protein expression was not be clearly assessed, and further researches were needed to elaborate.

## 5. Conclusion

In our study, we found that BolA gene family members (BOLA1-3) may serve as prognostic biomarkers of HCC. In addition, BolA family members and their neighborhood GLRX3 play a leading role in HCC stage and tumor grade. These interesting results have important implications that can identify novel therapeutic targets in HCC.

## Data Availability

All the data used to support the findings of this study are available online.

## Abbreviations

NP: Normal person
CHD: Coronary heart disease
CRC: Colorectal cancer
HCC: Hepatocellular carcinoma
PAAD: Pancreatic adenocarcinoma
WhB: Whole blood.

## Supplementary Materials

Supplementary Materials Figure S1: genetic alterations of 3 BolA family members were shown in HCC patients (cBioPortal). Notes: (a) OncoPrint of 3 BolA family member alterations in LIHC. (b) Using Exosomes web-accessible database (http://www.exoRBase.org) analysis, the increased expression of BOLA2 may be used as circulating biomarkers for HCC patients.
