# Supplementary material for: Prognostic Values of BolA Family Member Expression in Hepatocellular Carcinoma
Source: Biomed Res Int. 2022 Aug 16;2022:8360481. doi: 10.1155/2022/8360481 (PMC9398796; doi:10.1155/2022/8360481)
Supplement: Supplementary Materials — Figure S1: genetic alterations of 3 BolA family members were shown in HCC patients (cBioPortal). Notes: (a) OncoPrint of 3 BolA family member alterations in LIHC. (b) Using Exosomes web-accessible database (http://www.exoRBase.org) analysis, the increased expression of BOLA2 may be used as circulating biomarkers for HCC patients. [file 8360481.f1.docx]

**
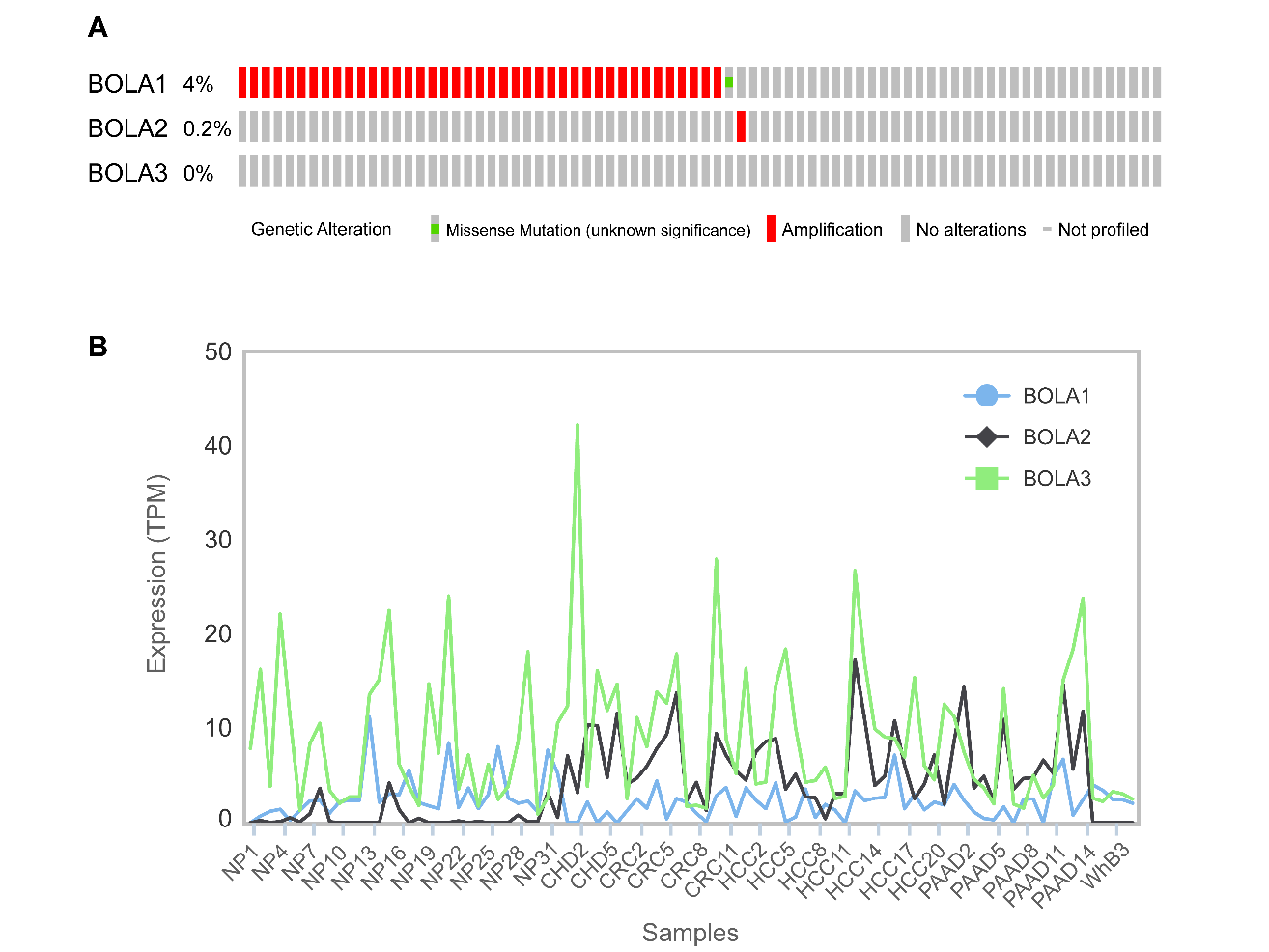
**

**Figure S1**. Genetic alterations of 3 BolAs family members were shown in HCC patients (cBioPortal). **Notes:** (**A**) OncoPrint of 3 BolAs family member alterations in LIHC. (**B**) Using Exosomes web-accessible database (<http://www.exoRBase.org>) analysis, increased expression of BOLA2 may be used as circulating biomarkers for HCC patients.

**Abbreviations:** NP, Normal person; CHD, Coronary heart disease; CRC, Colorectal cancer; HCC, Hepatocellular carcinoma; PAAD, Pancreatic adenocarcinoma; WhB, Whole blood.
